# Pathogenesis and treatment of wound healing in patients with diabetes after tooth extraction

**DOI:** 10.3389/fendo.2022.949535

**Published:** 2022-09-23

**Authors:** Shuting Yang, You Li, Chengcheng Liu, Yafei Wu, Zixin Wan, Daonan Shen

**Affiliations:** State Key Laboratory of Oral Diseases, National Clinical Research Center for Oral Diseases, West China Hospital of Stomatology, Sichuan University, Chengdu, China

**Keywords:** tooth extraction, diabetic, healing, dental extraction sockets, insulin-dependent diabetic

## Abstract

Diabetes mellitus is a common systematic chronic disease amongst dental patients. The elevated glucose microenvironment can prolong the healing of tooth extraction sockets. Therefore, the promotion of healing up tooth extraction sockets is of great clinical importance to the patients with diabetes mellitus. The current evidence indicates the mechanism of the recovery period of extraction sockets in hyperglycaemia conditions from physiological, inflammation, immune, endocrine and neural aspects. New advancements have been made in varied curative approaches and drugs in the management of wound healing of tooth extraction sockets in diabetes. However, most of the interventions are still in the stage of animal experiments, and whether it can be put into clinical application still needs further explorations. Specifically, our work showed topical administration of plasma-rich growth factor, advanced platelet-rich fibrin, leukocyte- and platelet-rich fibrin and hyaluronic acid as well as maxillary immediate complete denture is regarded as a promising approach for clinical management of diabetic patients requiring extractions. Overall, recent studies present a blueprint for new advances in novel and effective approaches for this worldwide health ailment and tooth extraction sockets healing.

## Introduction

Diabetes mellitus (DM) is recognized as an enormous menace to the general population globally, which affects 463 million adults (1). It is a systematic metabolic disorder characterized by defective insulin secretion and impaired insulin, resulting in microvascular complications and hyperglcemia (2). Diabetes is divided into diabetes mellitus type 1 (T1DM) and diabetes mellitus type 2 (T2DM), with T2DM making up 90% of cases worldwide and thus more relevant research (3). Patients with DM are associated with a high risk of hyperlipidemia, obesity, and healing disorders. Considering that diabetes ranks 3th in the most prevalent chronic disease in the oral field (4), number of diabetic patients experiencing oral manifestations exceeded 90% (5). Diabetic patients have a prevalence of missing teeth, prolonged wound healing, xerostomia, caries, burning mouth disorder, lichen planus, and even bacterial osteomyelitis of the jaw, which could increase the treatment difficulty and compromise the treatment outcome of various oral diseases (6–13). A population-based cohort study proved that diabetic patients have a higher risk of tooth extraction due to periodontal disease than non-diabetic patients in South Korea (p <.01) (6). The origin of the medication-related osteonecrosis of the jaws tends to be tooth extraction in elderly patients with uncontrolled diabetes (P < 0.0125) (14). Case reports proved that bacteraemia and fungal infection caused by diabetes-related tooth extraction seem to be a triggering factor for osteomyelitis and mucormycosis, respectively (15, 16).Therefore, elucidating the mechanism and investigating the approaches to promoting the healing of tooth extraction sockets is of great clinical importance, especially for the patients with DM. In this review, we systematically searched and appraised the current literature to summarize and discuss the mechanisms and managements of delayed extraction sockets in patients with diabetes.

## Mechanistic insight into delayed tooth extraction socket healing among diabetic patients

**Figure 1:**
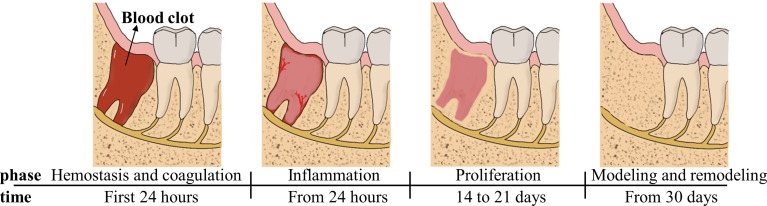
Main processes of wound healing occurring in the socket after tooth extraction depicted as four time-related phases.

The histological healing process in extraction-sockets is a four-stage process involving the blood clot phase, the inflammation phase of granulation tissue formation, the proliferation phase with woven bone formation and the modeling and remodeling phase, as shown below (**Figure 1**) (17, 18). Osteogenic tissue proliferates and bone maturity following trabecular bone formation occurs between 4 and 8 weeks after extraction (19, 20).

Delayed tooth extraction socket (TES) healing were often found in patients with poorly controlled or untreated DM (21). Tooth extraction healing is slower for diabetic than the group without diabetes, particularly on day 7 post-operatively (22). However, not all studies have reached the conclusion that diabetics have increased delayed healing (23). In the study by Goss et al. there was no statistically significant difference in healing rate after tooth extraction in either T1DM or T2DM compared to non-diabetic patients, a result that supports the tendency for diabetic patients to recover well after tooth extraction when they are well controlled (24, 25). For instance it has been shown that the duration of bone healing is similar in diabetic and normal individuals (24). Still, due to the specificity of diabetes and the possibility of delayed-wound-healing risk after tooth extraction, it is of great value to understand the mechanisms involved and the potential treatments.

**Figure 2:**
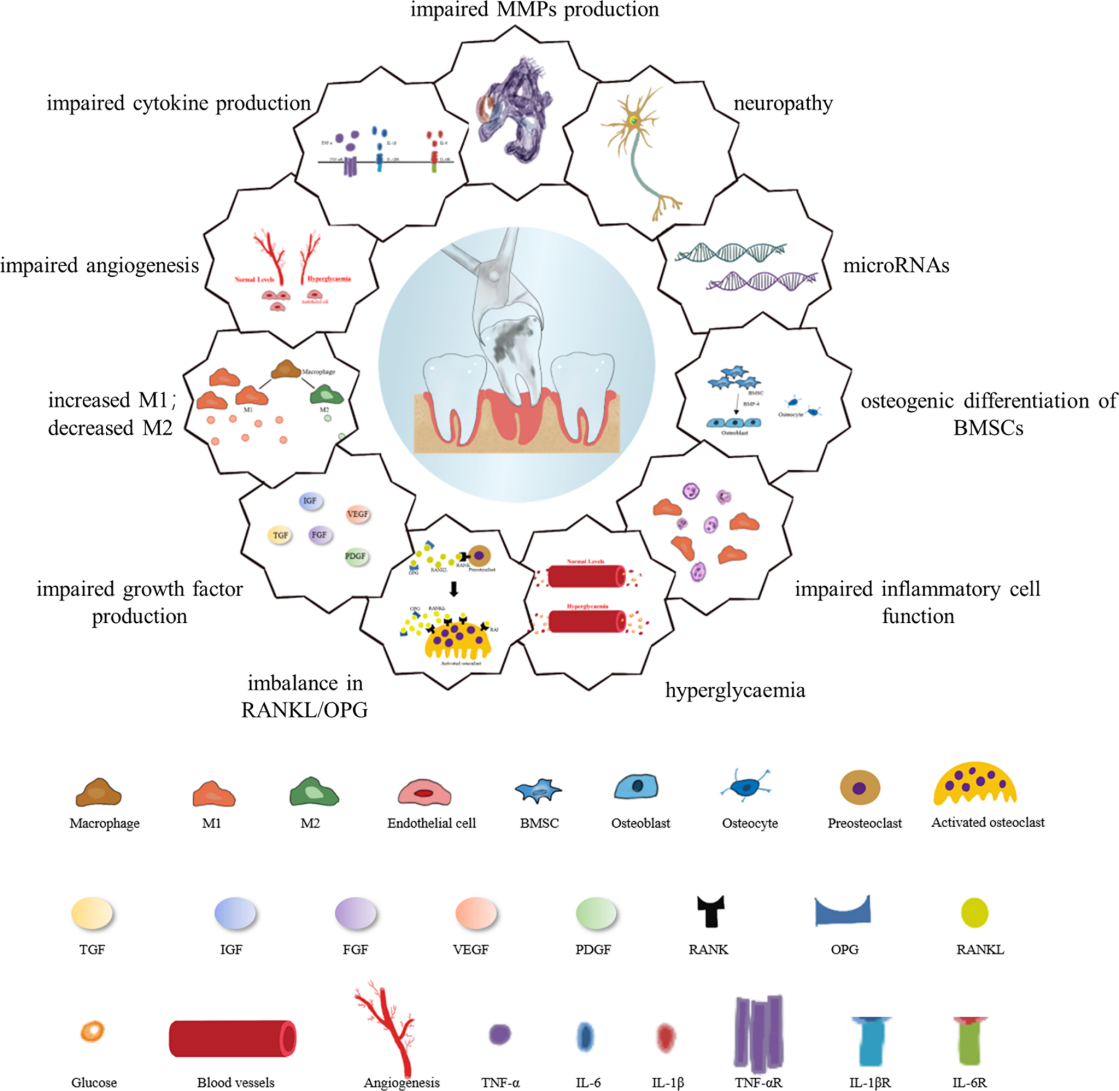
Factors responsible for the healing process of diabetic extraction sockets. Diabetes inhibits mitotic growth factor expression through epigenetic mechanisms; difficulty in wound healing after tooth extraction is associated with diminished osteogenic differentiation of mesenchymal stem cells, activation of matrix metalloproteinase-9, persistent imbalance of RANKL/OPG ratio, and reduced expression of neuropeptides. Hyperglycemia affects hormone receptor conversion as well as the formation of new blood vessels, and impaired angiogenesis not only hinders bone formation but also affects the rate of wound healing. Diabetic wounds are characterized by chronic inflammation due to high levels of reactive oxygen species, dysregulated M1/M2 macrophage polarization, and pro-inflammatory chemokines. High glucose levels have a negative impact on macrophage function, mainly in the form of dysregulated levels of cytokine secretion such as TNF-α, IL-6 and IL-1β, in addition to the inability of neutrophils to function in the inflammatory response phases of wound healing, migration, chemotaxis and adhesion. MicroRNAs also influence the different phases of diabetic wound healing.

In recent years, the field of wound research has been broadened by an in-depth understanding of diabetes and its various aspects of physiological, inflammatory, immunological, endocrine, neurological mechanisms and microRNAs (miRNAs) associated with the healing of extracted tooth sockets (26). Long-standing wound healing in patients with diabetes is generally attributed to the abnormal expression of all the cells involved as well as the dysregulation of the expression of growth factors, cytokines required to coordinate the normal healing process as suggested by these research. Factors accounting for the healing process of diabetic extraction sockets is presented in **Figure 2**.

### Physiological mechanism

Healing of extraction sockets is a complex process involving the reconstruction of damaged soft and hard tissues. It embodies the proliferation and differentiation of osteocytes, as well as the synthesis and mineralization of extracellular matrix, resulting in bone formation and remodelling. These activities are regulated by various cytokines, comprising the transforming growth factor β (TGFΒ), the vascular endothelial growth factor (VEGF), the insulin-like growth factor (IGF) and the bone morphogenetic protein (BMP) (27). The increased recovery rate was observed through the local application of growth factors; however, the deficiency of growth factors in hyperglycaemia conditions caused a low level of wound healing in animal or clinical studies (28, 29). Decreased expression levels of these TGFΒ1-3, TGFβRII and TGFβRIII genes may be linked to impaired oral mucosa healing in diabetic mice (30). Diabetes-induced detrimental effects on TES healing under the palatal plate may be mitigated due to the rise in salivary VEGF elicited by T2DM in clinical trials (31). However, the presence of VEGF would be insufficient to produce new bone under hyperglycemic conditions. The bone formation is disrupted due to crosslinking of advanced glycation end products (AGEs) unfavorably, in spite of induction of VEGF-C and VEGF receptor-3 positivity in Akita mouse osteoblasts after extraction (19). IGF-1 could foster the osteogenic differentiation of apical papillae stem cells, which is likely to be induced by c-Jun N-terminal kinase and p38 mitogen-activated protein kinase signaling pathways (32). In addition, the concentration alterations in tissue growth factors, such as IGF-1, may be strongly correlated with wound healing of the epithelium in rats (33). Noticeably, non-enzymatic glycosylation of collagen in hyperglycaemic rats was found to impair the collagen metabolism, thus producing highly soluble and easily degradable collagen. In this case, the mechanical properties of the formed bone were weakened, and led to the delayed healing and increased alveolar destruction (34).

Interestingly, the gene expression profile of T2DM was distinguishable from control subjects (35). According to Liang et al. (36), 11 differentially expressed genes were substantially higher in the non-diabetic control group than in the T2DM group, and among these genes, BMP-4, which is significantly under-expressed in T2DM blood, is the most important gene regulating bone marrow mesenchymal stromal cells (MSCs) osteogenic differentiation based on gene ontology annotation and random forest analysis. Among BMP family, BMP-4 was shown bone-forming potential in rat tooth sockets (37). BMP-4, associated with bone morphogenetic protein receptor 1, enhances the osteogenic differentiation of stem cells *via* activation of Smad signaling (38). It is noteworthy that recombinant BMP4/7 has a higher potential to induce MSC differentiation than BMP4 (39). With high concentrations of glucose (25mmol/l), the levels of BMP-4, bone sialoprotein and osteopontin expression, expression of Shh and alkaline phosphatase (ALP) were greatly reduced compared with low glucose (5.5mmol/l) (40).

The nature of diabetic wounds that are resistant to healing is also connected to the involvement of matrix metalloproteinase (MMP). The higher activity of MMP-2 and MMP-9 in diabetic mice wounds is similar to that of hard-to-heal wounds caused by ulcers or burns (41), and subsequently studies have identified MMP-8 and MMP-9 from diabetic wounds and demonstrated that MMP-8 inhibits apoptosis and favors wound healing, while conversely MMP-9 promotes apoptosis and renders wounds unhealable in mice (42). Infection of wounds increases MMP-9 activity, facilitates macrophage infiltration and diminishes angiogenesis in animal and clinical experiments (43). Selective inhibition of MMP-9 together with locally applied active recombinant MMP-8 supports wound healing in diabetes in mice (44). Hyperglycaemia (25mmol/L) can affect the regulation of cellular Na+/K+ adenosine triphosphate enzyme activity, increase protein kinase C activity, influence the conversion of hormone receptors and the formation of new blood vessels *in vitro* (45, 46). This is corroborated by the fact that lower ATP concentrations in plasma are coupled with lower blood flow in T2DM patients compared to healthy subjects (47). High blood glucose (>13.9mmol/L) can also contribute to the production of AGEs as well as receptor for AGEs (RAGE) under metabolic disorders and inflammatory conditions in diabetic rats (48). In *in vitro* experiments, increased AGE levels elevate extracellular MMP inducer content and stimulate the secretion of MMP, accompanied by collagen degradation and a decrease in bone strength (49). Large amount of aldoses of AGEs has been found to cause dysfunction of the endothelial cells and extracellular matrix of the microvascular wall by covalently bounding to active amino groups, and damage blood vessels by up-grading oxidative stress and inducing monocytes to produce platelet-derived growth factors (50). Thus the blood vessels became pathologically permeable and inelastic, and block the blood flow (51).

Receptor activator of nuclear factor kappa B (RANK) and its ligand (RANKL), as well as the deceptive receptor osteoprotegerin (OPG), are the three main proteins of the RANKL/RANK/OPG signaling pathway encoded by TNFRSF11B (52). RANKL-RANK interaction increases osteoclast production, whereas OPG inhibits their binding. This pathway is famed for its roles in bone remodeling and may have an impact on the pathogenesis of T2DM women (53). For poorly controlled T2DM patients, a continual imbalance in RANKL/OPG ratio may be produced in periodontal tissues (54).

Angiogenesis is described as new vessel formation out of pre-existing ones and exerts its effects on wound healing (55). The functional vascular supply is responsible for proper ossification of newly deposited bone (56). Impaired angiogenesis in patients with hyperglycaemia affects the rate of wound healing, in addition to impeding bone formation (57). Over and above that, hypoxia-inducible factor 1α may stimulate angiogenesis and enhance new bone formation as a transcription factor *in vitro* (58). During bone repair, its expression is upregulated due to hypoxia, but its function of mediating angiogenesis and osteogenesis is suppressed due to high glucose conditions in diabetic mice (59).

### Inflammation and immune mechanism

Alterations in inflammation levels and reductions in new connective tissue and bone formation played an essential role in diabetic oral wound healing. Diabetes suppresses mitogenic growth factor expression and increases pro-inflammatory cytokine expression mediated by epigenetic mechanisms (60). Chronic diabetic wounds are chronically inflamed due to a great deal of reactive oxygen species (ROS), dysregulated M1 macrophage polarization and pro-inflammatory chemokines in mice (61). TNF-α is acknowledged to stimulate inflammatory response by increasing the number of blood vessels and vessel density and regulating M1/M2 macrophage polarization in *in vitro* and animal studies (62–64). However, it is stated that elevated TNF-α and promoting inflammatory cytokines in a hyperglycemic state (>16.7mmol/L) instead spur bone resorption on the one hand and restrain bone formation on the other hand in rats (65). Runx2, important for the differentiation of osteoblasts and maturation of chondrocytes, is inhibited by pro-inflammatory cytokines *in vitro* (66, 67). A reduction of Runx2 diminished MSC differentiation and the production of osteoblast cells (68). Besides, granulocytes were unable to function during the inflammatory response stage of the wound healing, migration, chemotaxis and the adhesion of neutrophil in T1DM patients (69). The impaired neutrophil function, however, was found not related to the increased risk of short-term postoperative complications in T2DM (70). No correlation was found between extended wound epithelialization and reduced neutrophil function at three weeks postoperatively (70).

Uncontrolled DM patients are regarded as immunosuppressed, considering the negative impact of hyperglycaemia on the immune system. It has been confirmed that high blood sugar (25mmol/L) causes damage to the cellular immune response, inflammatory cytokines and microcirculation during the healing process (71, 72). The mechanism of impaired immune system is mainly related to immune cells, such as macrophages and granulocytes. High glucose levels (>16.7mmol/L) have a negative impact on the function of macrophages, mainly in the form of dysregulated secretion levels of cytokines such as TNF-α, IL-6 and IL-10, and decreased metabolic activity in T1DM mice (73); in combination with enhanced pro-inflammatory macrophages was found in T1DM mice in *in vitro* experiments, resulting in a higher risk of infection (74). Defects in phagocytosis may interfere with the inflammatory response and microbial uptake, causing accumulated debris in the wound and preventing the formation of granulation tissues both *in vivo* and *in vitro* (75). Notably, abnormal inflammatory responses coordinated by M1 or M2 macrophages are also usually associated with delayed healing. In a vitro co-culture model, M1 pro-inflammatory macrophages were found to act primarily by inhibiting capability of MSCs as well as the angiogenic ability of endothelial cells, while the opposite role was found for M2 anti-inflammatory macrophages (76). High glucose (25mmol/L) could drive M1 macrophage polarization *via* overproducing ROS under inflammatory stimulation in T1DM rats (77). The polarization of elevated M1 and reduced M2 macrophage may take responsibility for slowed TES healing in subjects with T2DM through aberrant expression of tumor necrosis factor-α (TNF-α) and peroxisome proliferator-activated receptor-γ (21).

### Endocrine mechanism and neural mechanism

In diabetic patients, their hyperglycemic condition affects a wide range of cell functions, for example, the regulation of bone-forming differentiation. Osteoblast proliferation and differentiation can be inhibited by hyperglycemia (25mmol/L) through caspase-1-mediated pyroptosis *in vivo* and *in vitro* (78). It may cause osteoblast bone formation disorders and result in pathological changes, such as diminished bone formation and reduced alveolar bone height of tooth extraction wounds. It has been suggested that the increased expression of glucose transporter 1 might be part of the reasons for the inadequate mineralization of osteoblasts during hyperglycaemia *in vitro* (79). Excessive protein-linkedΒ-acetylglucosamine glycosylation (-GlcNAcylation) induced by O-GlcNAc transferasewith high glucose (46,60 mmol/L), glucosamine (2.5-5mmol/L) or N-acetylglucosamine (5mmol/L) leads to a reduction in RUNX2 gene expression and thus has an inhibitory effect on osteogenic differentiation *in vitro* (80). Furthermore, the weakening of MSC osteogenic differentiation might be an essential factor responsible for TES healing for T2DM pig models (81). Growth differentiation factor 11 was related to the inhibited osteogenic differentiation of MSCs in TES in patients with T2DM (82).

Sensory nerves contribute to inflammation and immune response, in particular, possess trophic-facilitating wound healing generally (83). Neuropeptides are neuromodulators involved in a variety of processes, diabetic wound healing released by sensory nerves included (84). Insufficiency of neurogenic mediators such as substance P (SP), secreted from sensory neurons, may participate in wound epithelization in mutant diabetic mice with delayed healing (85). Moreover, SP stimulates bone formation in osteoblasts by neurokinin-1 receptors at advanced stages of bone formation in rats (86). Diabetes can lead to autonomic and small sensory nerve fiber neuropathy and dysregulation of inflammation, as evidenced by reduced expression of neuropeptide and imbalance in pro- and anti-inflammatory cytokine responses (87). It has been found that the exogenous SP improved wound repair kinetics and suggested that the chronic trauma in DM patients may be attributable to downgraded levels of neuropeptide nutrition (85).

### The function of microRNAs in the healing of diabetic wound

MiRNAs, regulating expression of mRNA, are a kind of short non-coding single-stranded RNA molecules (88). MiRNAs influence several physiological and pathological processes, the most notable being metabolism, proliferation, differentiation and apoptosis. Therefore, they are being investigated as vital markers at different stages of the wound healing process (89).

There are several miRNAs involved in the regulation of inflammatory phase of wound healing in a hyperglycemic environment. For example, inflammation in unhealed wounds of patients with T2DM affects plasma miRNA concentrations, whereas miR-191 affects angiogenesis through its target zonula occludens-1 in order to slow down the tissue reparative process (90). MiR-497, with its down-regulation activity for pro-inflammatory cytokines, to such factors as TNF-α, IL-1β, IL-6, is considered as a promising curative factor for diabetic wound healing in mice (91). MiR-129-2-3p at wound sites in type 2 diabetic mice may expedite wound healing by mediating the function of neutrophils (92).

Other miRNAs participating in angiogenesis and remodeling stages consist of miR-15b, miR-20b, miR-21, etc. Both 15b and 200b can inflict impaired angiogenesis by repressing the expression of VEGF in diabetic mice (93). In diabetic mice, knockdown of miR-20b-5p was found to significantly potentiate wound repair and facilitate wound angiogenesis by regulating the Wnt9b/β-catenin signaling pathway (94). It has been shown that miR-21 expression is engaged in early healing of the incisor extraction sockets in mice (95). Strauss et al. demonstrated that miR-21 knockout mice had approximately 15% reduced bone formation in the mesial and coronal portions of the extraction socket compared to wild-type controls (95). MiR-27b was revealed to prompt wound healing by rescuing damaged angiogenic cells in T2DM mice (96). For pigs and mice, anti-angiogenic MiR-92a, its inhibitor, possesses the ability to accelerate wound healing (97). In *in vitro* experiments, upregulated MiR-140-3p exosomes promoted the differentiation of MSCs into osteoblasts (98).

Nevertheless, the study of miRNAs and diabetic TES healing paves the way for miRNA-based dental regeneration strategies.

## Potential interventions in the management of extraction sockets healing in patients with diabetes

Ideal interventions used in oral surgery should facilitate the repair of extraction sockets, and reduce the postoperative infection, pain and complications. Plenty of investigations have explored pathways to acceleration of TESs healing under high-glucose conditions based on molecular regulators of their activity, either directly or indirectly. It is encouraging to see that a considerable number of results have entered clinical trials, as shown in the table below (**Table 1**). Directly interacting targets include growth factors, BMPs, parathyroid Hormone (PTH), and stem cells. A variety of drugs may act indirectly on molecular targets by up- or down-regulating the expression of growth factors, MMP, collagen synthesis/degradation, pro- and anti-inflammatory cytokines, and pro-angiogenic factors. Drugs or natural products or formations of molecular targets that are involved on a direct or indirect basis in a proposed treatment will be described below.

**Table 1:** List of clinical trials studies on extraction sockets healing in patients with diabetes.

Intervention	Year	Study design	Results	Reference
PRGF	2014	Retrospective, split-mouth study	PRGF reduced residual socket volumes and improved Healing Indices	(99)
L-PRF	2019	Prospective, double-blind, split-mouth study	L-PRF enhanced bone density (p=0.007)	(100)
	2019	Prospective, randomized, double-blind, controlled study	L-PRF and HA mucosa improved healing scores within 3 weeks	(101)
A-PRF	2019	Randomized, split-mouth, double-blind Study	A-PRF slightly affected PD positively	(102)
HA	2020	Randomized controlled split-mouth study	The sockets healing was better in the HA group, especially on day 10 (p=0.006) and day 15 (p=0.021)	(103)
MICD	2016	Prospective study	MICD reduced SOD significantly and improved chewing ability within 3 weeks	(104)

L-PRF leukocyte- and platelet-rich fibrin, A-PRF advanced platelet-rich fibrin, HA hyaluronic acid, PD pocket depth, MICD maxillary immediate complete denture, SOD socket opening diameters

### Molecular targets

Local delivery of growth factors, as for instance by the delivery of platelet-derived growth factor (99), IGF (79), fibroblastic growth factor (105), has been verified to favor wound healing in poorly controlled diabetes. Systematic reviews and meta-analyses have found the efficacy of platelet derivatives to improve the wound healing and bone density, thereby stimulating the soft tissues and bone regeneration (106, 107). Platelet-rich plasma is dependent on platelets to exert great influence on healing. In a split-mouth study recruiting 34 patients with T1DM, the application of plasma-rich growth factor after extraction yielded remarkably diminished residual TES volumes and improved Healing Indices by accelerating the socket closure (epithelialization) and tissue maturation in diabetic patients (99). Another animal research study assessed the effect of topical application of autologous platelet-rich plasma on extraction wound and found that it prevents the medication-related osteonecrosis of the jaws (108). Activated platelet lysates induce OPG expression and stimulate soft tissue healing and osteoblast differentiation in rats (109). IGF-I was found to increase the volume of neoformed bone after tooth extraction in diabetic rats by regulating glucose transporter 1 expression, as well as increases osteoblast mineralization during extraction wound healing (79, 110). For those patients with insulin resistance, IGF-I treatment can be considered, but the effectiveness and safety of IGF-I for long-term use in the management of diabetes and complications involved need further studies (111). Hence, locally delivered growth factors to promote healing may be a potential therapy for the treatment of diabetic osteopathy.

Local hemostatics are beneficial in reducing underlying postsurgical bleeding and to pace healing (112). Leukocyte- and platelet-rich fibrin (L-PRF) enhanced bone density and reduced inflammation, used as a graft to fill the TES and stabilize the blood clot in patients (100). L-PRF alone or in combination with hyaluronic acid (HA) was effective in improving mucosal healing and preventing alveolar osteitis and infection following mandibular third molars extraction (101). However, it has also been the finding that L-PRF adds to the growth factors concentration in the TES but has no positive outcome on bone healing (113). Additionally, the finding demonstrated the potential of advanced platelet-rich fibrin (A-PRF) as a therapeutic biomaterial for bone regeneration after surgical extractions of third molars in clinical trials, but further studies with larger sample sizes and more systematic and reliable evaluation tools are necessary (102).

Treating the diabetic sockets with BMP may be useful to TES healing. Controlled local release of recombinant human BMP-2 dramatically promoted bone production in diabetic mice to near normality and potentiates bone rejuvenation in normal mice (114). BMP-6 can facilitate the osteoblast differentiation from MSCs and the chondrocyte maturation by signalling through type I and type II BMP receptors (115). The extra-alveolar tissue of diabetic rats showed a subcellular periosteal reaction by day 3, and a large amount of cartilage had been formed by day 7 following the application of BMP-6 (116). It has been reported that down-regulated BMP-6 in certain tissues such as myofibroblast progenitor cells in diabetic patients thereby inhibited the cartilage formation delaying the healing (117). Therefore, the topical application of BMP-6 is promising to reverse the healing inhibition of diabetes. Moreover, the level of expressed BMP-4, bone sialoprotein and osteopontin, ALP activity and the increased number of matrix mineralized nodules in MSCs correlated with the Lenti – Shh activated Shh signaling pathway; *in vivo* experiments revealed that Lenti – Shh invoked additional osteogenesis (40). The intraoral injection of the inhibitor of growth differentiation factor 11 has been found to promote the bone healing in the post-extraction site as well as the osteogenic differentiation of porcine MSCs (82). Furthermore, activating macrophages by mannose receptor clustering and enhancing M2 macrophage polarization were found to contribute to accelerated wound healing, increase the collagen expression and reduce the infection in hyperglycemic conditions in mice (118). Sustained Interleukin-4 released markedly enhanced osteogenic and angiogenic gene expression with improved socket healing in T2DM mice by inducing macrophage transformation towards M2 polarization (21).

PTH is an important hormone to regulate the bone metabolism. PTH has been shown to reduce the alveolar bone loss in the intermittent and systemic administrations by decreasing the RANKL/OPG ratio in diabetic rats (119). However, some studies found that PTH did not improve the post-extraction wound healing or stimulate the osseointegration in hyperglycemic rats, regardless of administration of PTH (intermittent versus continuous) (120). This can be explained by the overall inhibitory effect of high levels of AGEs and collagen cross-linking on bone formation under diabetic metabolism (121). The anabolic role of PTH in the repair after DM extraction needs to be confirmed by further studies.

### Synthetic drugs

The acceleration of TES recovery with insulin or metformin has already been reported in previous research (34, 122). Insulin, a first-line drug in the clinical therapy of DM, can directly hasten TES healing by raising TGFβ-3 expression and lowering IGF-1R expression in diabetic rabbits (27). Moreover, the consequences of high blood glucose and metformin on peri-implant healing should be attached importance to. Metformin is the most commonly employed oral hypoglycemic agents; its benefit attributed to its preferential influence on endothelial cells, as well as its antioxidant and anti-inflammatory properties (123). Metformin not only remarkably reduced both intracellular ROS and apoptosis, but also increased osteoblast differentiation at varied glucose levels (0.99, 1.98, 3.96, and 7.92 g/L), which may be related to the promotion of Runx2 and IGF-1 expression *in vitro* (122). Noticeably, osteogenic differentiation potential of MSCs could be enhanced by metformin in T2DM patients through the BMP-4/Smad/Runx2 signaling pathway (36). Goto-Kakizaki rats with T2DM showed improved blood glucose and bone volume percentage, the number of trabecular, as well as bone density after using metformin (124).

### Natural product-based treatment

Natural ingredients, namely obtained from natural sources, often stand for the topic of further research and have been exploited as an alternative therapy like spirulina, chitosan, flavonoids and many more. Chitosan is a deacetylated polysaccharide from chitin, which can accelerate new bone formation and enhance neovascularization *in vivo* (125). Besides chitosan, spirulina, a microalgae containing kaempferol, also has antioxidant and anti-inflammatory effects (126). Due to the fact that the addition of 12% spirulina and 20% chitosan to the dental socket of mice yielded an alkaline pH that was suited to ALP activity, the bone remodeling process can be completed by promoting an increase in osteoblast cells and a decrease in osteoclasts (127). Ellagic acid is a natural component that effectively prevents bone loss induced by tooth removal in diabetic rats; diabetic rats treated with ellagic acid express a stronger immunohistochemical response to fibroblastic growth factor-2 and ALP than non-treated diabetic rats (105).

Flavonoids are known as a natural component that can inhibit inflammation whilst speeding up wound healing. Morin, as a pleiotropic dietary flavonoid, may prevent bone histomorphological alterations in diabetic rats through a potential mechanism of the insulin/IGF-1 pathway (128). Extract of okra fruit containing flavonoid, possesses strong antioxidant and anti-inflammatory properties. Okra fruit extract (250 mg/kg) increased TGFΒ1 levels in post-extraction wounds of diabetic Wistar rats (129). Treatment of hyperglycemic diabetic rats with a new chemically modified curcumin 2.24 contributed to the alleviation of local and systemic inflammation and reduced bone loss, plus inhibition of collagenolytic MMPs as well as pro-inflammatory cytokines (130). A modified curcumin was found to accelerate skin wound healing in hyperglycemic rats induced by streptozotocin (131). Probiotics serve as a potential strategy to augment insulin sensitivity and minimize autoimmune responses by modifying intestinal flora and reducing inflammatory responses and oxidative stress (132). It is showed that exogenous SP favourably promotes wound healing kinetics in Mutant diabetic mice (85). Further, new bone formation was enhanced histomorphometrically when using deproteinized bovine bone mineral containing 10% collagen with hypoxia-inducible factor 1α in dogs (133). These materials provide a clue for latent auxiliary therapies in the management of post-extraction wound in patients with DM.

### Other approaches

HA could be a reliable approach to wound closure. One study investigated the underlying role of HA, a component of extracellular matrix, in promoting TES healing in diabetic patients. In a randomized controlled split-mouth study including 30 patients with poorly controlled T2DM who required tooth extraction, 0.8% HA placed in post-extraction socket improved the wound healing, in particular on the first days after applying (103). In addition, sodium hyaluronate (HY) is the product of the neutralization of the carboxyl groups of HA, which has been proved to enhance the healing process in the extraction sockets of rats (134). Diabetic rats gained greater percentage of newly formed trabeculae in the post-extraction wound treated with HY or carbon nanotubes functionalized with HY (135).

Low-level laser therapy offered a good treatment option for TES healing in T2DM patients (136). Rat sockets irradiated by 808 nm or 660 nm laser had less inflammatory cell infiltration and more angiogenesis than unirradiated sockets apparently (137). Low-level laser therapy at 808 nm was able to considerably improve osteoid regeneration, while no substantial difference was observed in the amount of bone formation with 660 nm (137). Park et al. agreed that 980-nm laser irradiation in diabetic and normal rats for 1 minute per day contributed to early TES healing and further calcification with a high expression of Runx2 and collagen type I mRNA (138). Maxillary immediate complete denture has been considered as a feasible treatment for TES healing in T2DM patients with lower reduction of socket opening diameters, as it offers an opportunity to train chewing ability, and thus maintaining good nutrition in post-extraction period (104).

To date, the clinically safe and effective therapy to facilitate the healing of TESs in patients with DM is still lacking. Many clinical trials and animal experiments have explored the interventions for facilitating the healing of extraction sockets and improving clinical symptoms (24, 105). However, the efficacy of these methods is not satisfactory because of the complicated nature of diabetes, the fragility of the oral environment and short-term assessment. Well-designed large-scale multi-centre clinical trials are still required for the investigation of interventional wound healing diabetics.

## Conclusions

This review investigated the mechanism and treatment of the extraction sockets healing process in diabetic patients. Approaches involving the growth factor, growth factors, BMP, PTH, stem cells, synthetic drugs, natural product, HA, Low-level laser therapy have been evaluated with limited achievement. Various clinical trials have been explored to enhance the healing process of post-extraction sockets under hyperglycemic conditions, including plasma-rich growth factor, L-PRF, A-PRF, HA, maxillary immediate complete denture. However, most of these interventions are mostly still in the stage of animal experiments, and further studies are still needed before they can be applied in clinical practices. In the light of these facts, they present a hope that new approaches development will further supervene for this worldwide health ailment and healing of tooth extraction sockets.

## Author contributions

SY: researched data, wrote, reviewed, and edited the manuscript. YL: commentary. YW, ZW and CL: revised the manuscript. DS: critical review, funding acquisition. All authors contributed to the article and approved the submitted version.

## Funding

This study was supported by the National Natural Science Foundation (82170970).

## Conflict of interest

The authors declare that the research was conducted in the absence of any commercial or financial relationships that could be construed as a potential conflict of interest.

## Publisher’s note

All claims expressed in this article are solely those of the authors and do not necessarily represent those of their affiliated organizations, or those of the publisher, the editors and the reviewers. Any product that may be evaluated in this article, or claim that may be made by its manufacturer, is not guaranteed or endorsed by the publisher.
